# Genome-wide identification and characterization of the *MsTIFY* gene family in *Malus sieversii* and their response to abiotic stresses

**DOI:** 10.3389/fpls.2026.1830560

**Published:** 2026-06-15

**Authors:** Yongfeng Su, LIjun Liu, Deen Zhang, Yuanyuan Jing, Xiaoyan Lu

**Affiliations:** Department of Horticulture, College of Agriculture, Shihezi University, Shihezi, China

**Keywords:** abiotic stress, expression pattern, *Malus sieversii*, *MsTIFY* gene family, subcellular localization

## Abstract

TIFY proteins are plant‑specific transcription factors characterized by a conserved TIFY domain (TIF[F/Y]XG) and are widely involved in responses to abiotic stresses. In this study, 57 *MsTIFY* members were identified at the whole‑genome level in *Malus sieversii*. Their coding sequence (CDS) lengths and protein physicochemical properties (protein length, pI, GRAVY, and molecular weight) were compiled. Phylogenetic analysis, motif organization, and conserved domains were examined to characterize the TIFY, JAZ, PPD, and ZIM subfamilies. Secondary structures (random coils, alpha helices, beta turns, extended strands) and subcellular localization were predicted. Chromosomal localization and collinearity analysis were performed to assess duplication events. Collinearity with four other plant species (*Salvinia cucullata, Juglans regia, Arabidopsis thaliana, Manihot esculenta*) was analyzed. Promoter sequence analysis and Gene Ontology (GO) functional annotation were conducted. Expression patterns under abiotic stresses were investigated using transcriptome and quantitative real‑time PCR (qRT‑PCR), and subcellular localization of *MsJAZ2* and *MsJAZ26* proteins was confirmed experimentally. *MsTIFY* genes were classified into TIFY, JAZ, PPD, and ZIM subfamilies. The secondary structures of *MsTIFY* proteins were primarily composed of random coils and alpha helices. Subcellular localization predictions indicated that *MsTIFY* members are mainly expressed in the nucleus, which was experimentally confirmed for *MsJAZ2* and *MsJAZ26*. Whole‑genome duplication and tandem duplication play significant roles in driving the evolution of *MsTIFY* genes. Collinearity analysis suggested that TIFY genes are widely conserved between gymnosperms and angiosperms. Promoter analysis and GO annotation revealed potential functions. Expression analysis showed that *MsTIFY* members exhibit broad responses to different abiotic stresses: 14 genes showed significantly altered expression under cold stress, four under salt stress, and 12 under drought stress. The findings of this study provide insight into the role of *TIFY* genes in the stress response of *Malus sieversii* and in apple molecular breeding.

## 1 Introduction

*Malus sieversii* is predominantly distributed across the Tianshan Mountains, which extend through China, Kazakhstan, Kyrgyzstan, and Uzbekistan (Volk et al., 2005; Yan et al., 2008). As one of the primary progenitors of the cultivated apple, *M. sieversii* has been shown to contribute approximately 46% of the nuclear genome of the domesticated apple (Sun et al., 2020; Velasco et al., 2010). Over the course of 3 million years of evolutionary history, *M. sieversii* has gradually developed excellent traits such as drought resistance and cold resistance (Da et al., 2019; Zhou et al., 2021). As a vital wild germplasm resource worldwide, *M. sieversii* is a key focus in the expansion of genetic diversity and the introduction of beneficial alleles into apple breeding programs (Zhang, 1973; Ha et al., 2021). The red-fleshed variety *Malus sieversii* f. *niedzwetzkyana* has been used as a key parental material in the breeding of high-flavonoid and red-fleshed apple cultivars (Wang et al., 2017a). A total of 258 accessions of *M. sieversii* germplasm resources originating from seven natural populations were subjected to inoculation with the fire blight pathogen *Erwinia amylovora*. Their resistance levels were systematically evaluated, and the resistant germplasm subsequently screened (Zhu et al., 2025). As an apple rootstock, *M. sieversii* has a well-developed root system and demonstrates multiple advantageous traits. These include vigorous growth, cold tolerance, drought resistance, tolerance to poor and saline–alkali soils, strong graft compatibility with cultivated apples, rapid seedling emergence, and a short nursery cycle (Xing, 2024). Consequently, investigating the response mechanisms of *M. sieversii* to abiotic stresses, such as low temperature, drought, and salinity, is critically important to enhance stress resistance and promote environmental sustainability in apple production.

As transcription factors, proteins bind to specific DNA sequences (*cis*-acting elements) to activate or repress the transcription of downstream target genes, thereby precisely orchestrating plant growth, development, and environmental responses (Zhou et al., 2008). The *MsDREB6.2* gene of *M. sieversii* is significantly induced by drought and salt stress, and overexpression of *MsDREB6.2* markedly enhances the drought and salt tolerance in *Arabidopsis* plants (Liao, 2016). The *TIFY* family is a plant-specific transcription factor gene family that contains a conserved TIFY domain (TIF[F/Y]XG) (Vanholme et al., 2007). The *TIFY* family was originally designated as the *ZIM* gene family but was later renamed based on its characteristic TIFY domain (TIF[F/Y]XG) (Nishii et al., 2000; Vanholme et al., 2007). It is divided into four sub-families based on their conserved sequence characteristics: the TIFY domain (TIF[F/Y]XG), the JAZ domain (JASMONATE ZIM domain), the ZML domain (zinc finger inflorescence meristem: ZIM and ZIM-like), and the PPD domain (PEAPOD) (Bai et al., 2011). *TIFY* family genes play pivotal roles in the regulation of plant development and in the response to abiotic stresses (Gupta et al., 2021). As the first *TIFY* family member identified in *Arabidopsis*, *AtTIFY1* plays a role in inflorescence development and flowering while also promoting petiole and hypocotyl elongation through the mediation of cell elongation (Shikata et al., 2004). *OsTIFY11b* increases the plant height and seed size in rice (*Oryza sativa*), while *OsTIFY11a* forms a complex with *OsbHLH* and *OsNINJA* to regulate salt tolerance in rice (*O. sativa*) (Hakata et al., 2012; Ye et al., 2009). *TdTIFY11a* enhances the germination rate of wheat under saline conditions (Ebel et al., 2018). *GsTIFY10* is induced by bicarbonate, salinity stress, and the phytohormone jasmonic acid (JA) in both the leaves and roots of *Glycine soja*. Overexpression of this gene in *Arabidopsis* results in enhanced tolerance to bicarbonate stress during seed germination and the early and adult seedling developmental stages (Zhu et al., 2011). *GmTIFY10e* and *GmTIFY10g* are significantly upregulated under salt stress. Their overexpression enhances salt tolerance in both *Arabidopsis* and soybean (*Glycine max*) (Liu et al., 2022). Cold-upregulated *CaTIFY7* and *CaTIFY10b* enhance pepper cold tolerance by promoting cold-induced and reactive oxygen species (ROS)-related gene expression, while silencing them has the opposite effect (Wang et al., 2023). The *PnJAZ1* gene was cloned from *Pohlia nutans* and contains a conserved JAZ domain (JASMONATE ZIM domain). Overexpression of this gene enhances salt tolerance in *Arabidopsis* and *Physcomitrella* plants, reduces their sensitivity to abscisic acid (ABA) during seed germination, and suppresses the expression of the ABA signaling pathway-related genes (Liu et al., 2019).

With the advancement of sequencing technologies, an increasing number of species have undergone whole-genome sequencing, and their genomic data have been made publicly available. To date, genome-wide identification and characterization of the *TIFY* gene family have been performed in multiple species. For instance, 20 members were identified in rice (*Oryza sativa*) (Ye et al., 2009), 24 members in poplar (*Populus trichocarpa*) (Wang et al., 2017b), 30 members in maize (*Zea mays*) (Zhang et al., 2015), 34 members in soybean (*Glycine max*) (Zhu et al., 2013), and 49 members in wheat (*Triticum aestivum*) (Ebel et al., 2018). Despite the systematic identification of the *TIFY* gene family in the crops listed above, related research has predominantly focused on herbaceous plants and certain model woody species. In contrast, *M. sieversii*, which serves as the wild progenitor of apple and constitutes a crucial germplasm resource with robust stress resistance, has yet to undergo a systematic investigation concerning the genome-wide identification, evolutionary characteristics, and abiotic stress-related functions of its *TIFY* gene family.

In this study, based on the whole-genome data of *M. sieversii*, members of the *MsTIFY* gene family were identified. We analyzed their physicochemical properties, gene structures, and phylogenetic relationships. Subcellular localization was predicted for all family members, with experimental validation conducted for selected ones. The transcriptional regulatory potential of *MsTIFY* genes was predicted through analysis of the *cis*-acting elements. The chromosomal distribution and the expansion mechanisms of the *MsTIFY* gene family were explored by integrating chromosomal localization and collinearity analysis. Potential functions of *MsTIFY* genes were predicted based on Gene Ontology (GO) annotation. Furthermore, by integrating transcriptome sequencing and quantitative real-time PCR (qRT-PCR) experiments, the expression patterns of these family members under cold, drought, and salt stress conditions were determined. These results provide a foundation for the in-depth functional characterization of the *MsTIFY* genes in *M. sieversii*.

## 2 Materials and methods

### 2.1 Plant materials and growth conditions

*M. sieversii* plantlets were used in this study. They originated from the proliferation of a single plant line from prior work (He, 2020). The single plant line was cultured on a proliferation medium for multiplication and then transferred to a rooting medium for root induction. After 60 days, uniformly grown tissue-cultured plantlets were selected for subsequent experiments. The basal Murashige and Skoog (MS) medium contained 30 g L^−1^ sucrose, 6.4 g L^−1^ agar, and 4.4 g L^−1^ MS. The shoot proliferation medium was MS supplemented with 0.5 mg L^−1^ indole-3-butyric acid (IBA) and 2 mg L^−1^ 6-benzylaminopurine (6-BA), while the rooting medium consisted of 1/2 MS with 0.5 mg L^−1^ IBA. All media were autoclaved at 121°C for 20 min and stored aseptically under cold stress. Both seedlings and tissue-cultured plantlets were grown in an artificial climate chamber (RXZ Intelligent, Ningbo Jiangnan Instrument Factory, Ningbo, China) under the following conditions: 5,000 lx light intensity, a 14-h/10-h (light/dark) photoperiod, temperatures of 25°C (day) and 23°C (night), and 75% relative humidity.

### 2.2 Treatment of materials

#### 2.2.1 Cold stress

After rooting, the tissue-cultured plantlets with six to eight leaves were used for the experiment, with materials cultured under normal temperature serving as the control (CK). The treatment conditions were as follows: the temperature was lowered from 25°C to −3°C at a rate of 4°C h^−1^. The time when −3°C was reached was designated as 0 h, and the plantlets were then maintained at −3°C for 3, 6, and 12 h. The experimental materials were treated in a modified refrigerator (Rongsheng BD/BC-310 MS, Hisense, Gongdong, China) under the following controlled conditions: light intensity of 5,000 lx, a photoperiod of 25°C for 14 h (day) and 23°C for 10 h (night), and a relative humidity of 75%. Both the treatment groups and the CK group were set up with three biological replicates (Ma, 2022).

#### 2.2.2 Salt stress

Following rooting, the tissue-cultured seedlings (six to eight leaves) were subjected to 1) CK with an MS nutrient solution and 2) 150 mM NaCl treatment, with the NaCl concentration increased stepwise by 50 mM daily until all treatments simultaneously reached the target concentration (designated as 0 h). The leaves and roots of *M. sieversii* seedlings were collected at 6 and 48 h of treatment: controls were designated as LCK (leaves) and RCK (roots), while NaCl-treated leaves were labeled L6h and L48h and NaCl-treated roots as R48h (He, 2020).

#### 2.2.3 Drought stress

After rooting, tissue-cultured plantlets with six to eight leaves were treated as follows: the CK group consisted of an MS liquid medium without polyethylene glycol 6000 (PEG 6000). For the drought treatment, drought stress was simulated using an MS liquid medium containing 30% (*w*/*v*) PEG 6000. Hydroponically cultured seedlings were grown in an MS medium containing 30% PEG 6000 for 3 days. Control leaves and roots were labeled LCK and RCK, respectively, while the leaves and roots from the PEG treatment were labeled LPE and RPE, respectively (Li et al., 2019).

### 2.3 Identification and physicochemical characterization of *MsTIFY* genes

The conserved TIFY domain (PF06200) was obtained from the Pfam database (http://pfam.xfam.org/). The TIFY domain was used to search the *M. sieversii* protein database (https://www.rosaceae.org/Analysis/10816132) (Sun et al., 2020) with HMMER 3.1 (http://hmmer.org/download.html) (Mistry et al., 2013) to identify candidate sequences that contain the domain. Furthermore, the 18 known AtTIFY protein sequences were used as queries in a BLASTP search (*E*-value < 1 × 10^−5^) against the *M. sieversii* genome to acquire additional candidate *MsTIFY* gene members (**Supplementary Table 7**). All candidate sequences were validated for the presence of the TIFY domain with the SMART (http://smart.embl-heidelberg.de) and NCBI-CDD (http://www.ncbi.nlm.nih.gov/Structure/cdd/wrpsb.cgi) databases. The physicochemical properties of the identified MsTIFY proteins, including amino acid number, molecular weight (MW), and theoretical isoelectric point (p*I*) (**Supplementary Table 1**), were analyzed with the online ExPASy ProtParam tool (https://www.expasy.org/) (Wilkins et al., 1999).

### 2.4 Phylogenetic and motif analysis of *MsTIFY* genes

Conserved motifs within the MsTIFY protein sequences were identified using the online tool MEME Suite v5.5.6 (Bailey et al., 2009). The analysis parameters were set as follows: the maximum number of motifs to search (-nmotifs) was 10, and the motif width range (-minw and -maxw) was 6–50 amino acid residues. Only motifs with an *E*-value <0.001 were considered significant. A multiple sequence alignment of the identified MsTIFY proteins and 18 known *Arabidopsis thaliana* AtTIFY proteins was conducted using the ClustalW algorithm (Larkin et al., 2007) integrated in MEGA 7.0 (Kumar et al., 2016). All ClustalW parameters were kept at their default values: gap opening penalty = 10.0, gap extension penalty = 0.2, protein weight matrix = Gonnet, and delay divergent cutoff = 30%. Predefined gaps were not retained. Based on the alignment, an unrooted phylogenetic tree was constructed in the same software using the neighbor-joining method. Evolutionary distances were estimated using the JTT (Jones–Taylor–Thornton) model, and gaps/missing data were treated by pairwise deletion. The reliability of the tree branches was assessed using 1,000 bootstrap replicates. The phylogenetic tree was visualized with TBtools v2.451 (Chen et al., 2023).

### 2.5 Subcellular localization and secondary structure prediction

The secondary structures of the MsTIFY proteins were predicted with SOPMA (https://npsa-prabi.ibcp.fr/cgi-bin/npsa_automat.pl?page=/NPSA/npsa_sopma.html) (Geourjon and Deléage, 1995). Subcellular localization predictions were performed with WoLF PSORT (https://wolfpsort.hgc.jp/) (Horton et al., 2007).

### 2.6 Promoter *cis*-acting element analysis and GO annotation

The promoter regions (2,000 bp upstream of the start codon) of the *MsTIFY* gene start codons were extracted from the *M. sieversii* genome database (https://www.rosaceae.org/Analysis/10816132) (Sun et al., 2020) with TBtools. Potential *cis*-acting elements were identified in the promoter regions of the *MsTIFY* genes in the Plant CARE database (http://bioinformatics.psb.ugent.be/webtools/plantcare/html/). The protein sequences of the *MsTIFY* genes were submitted to the online tool eggnog mapper (https://eggnog6.embl.de/app/seqscan/) to identify the GO terms associated with the MsTIFY proteins.

### 2.7 Chromosomal localization, collinearity analysis, and selection pressure calculation of the *MsTIFY* genes in *Malus sieversii*

Chromosomal length information was extracted from the GFF3 annotation file of *M. sieversii*. The positional information of *MsTIFY* genes was retrieved using TBtools v2.451 (Chen et al., 2023) and then imported into Advanced Circos to generate a chromosome distribution map. For intra-species collinearity analysis, the “One Step MCScanX” function of TBtools was employed. Whole-genome protein sequences were subjected to self-BLAST (*E*-value ≤ 1*e*−5), and MCScanX (Wang et al., 2012) was run in combination with the GFF3 annotation. Collinear pairs involving *MsTIFY* genes were filtered and visualized. For inter-species collinearity analysis, *Arabidopsis thaliana*, *Juglans regia*, *Manihot esculenta*, and *Salvinia cucullata* were selected. Bidirectional BLASTP searches (*E*-value = 1*e*−5) were performed between *M. sieversii* and each of the four species. The resulting alignment files were merged and used as input for MCScanX to identify collinear pairs containing *TIFY* genes. The coding sequences (CDS) (**Supplementary Table 7**) of the identified collinear gene pairs were submitted to the “Simple *K*_a_/*K*_s_ Calculator” in TBtools to compute the non-synonymous substitution rate (*K*_a_) and the synonymous substitution rate (*K*_s_) (Zhang et al., 2006). The *K*_a_/*K*_s_ ratio was used to infer selection pressure (*K*_a_/*K*_s_ < 1 indicates purifying selection, while *K*_a_/*K*_s_ > 1 indicates positive selection).

### 2.8 Subcellular localization of *MsJAZ2* and *MsJAZ26*

The *pBI121–MsJAZ2–GFP* and *pBI121–MsJAZ26–GFP* fusion expression vectors were generated. The recombinant constructs *pBI121–MsJAZ2–GFP*, *pBI121–MsJAZ26–GFP*, and *AtHY5–CHERRY* (a red fluorescent nuclear marker) were transformed into GV3101 competent cells. GV3101 strains carrying these vectors were combined at a 1:1 ratio (*pBI121–MsJAZ2–GFP* with *AtHY5–CHERRY* and *pBI121–MsJAZ26–GFP* with *AtHY5–CHERRY*), and the mixtures were infiltrated into *Nicotiana benthamiana* leaves. The infiltrated leaves were kept in darkness for 24–36 h. Fluorescence signals were visualized using a confocal laser scanning microscope (LSM 800; Zeiss, Oberkochen, Germany) at excitation wavelengths of 488 nm (green fluorescent protein, GFP), 561 nm (red fluorescence), and 681 nm (chloroplast autofluorescence). The primers employed for gene cloning and vector construction are provided in **Supplementary Table 20**.

### 2.9 Expression analysis under cold stress and salt stress

Based on our group’s previous transcriptomic data under cold stress (Ma, 2022) and salt stress (He, 2020), gene expression dispersion was estimated using a negative binomial distribution model, followed by data normalization, and differential expression analysis was performed for all genes (**Supplementary Table 6**). Differentially expressed genes (DEGs) were screened using the following criteria: |fold change| ≥ 1.5 or |fold change| ≤ 0.5 and an adjusted *p*-value (*p*_adj_) <0.05. From these DEGs, the expression data of the corresponding genes were extracted according to the sequence IDs of the 57 identified *MsTIFY* gene family members. Subsequently, *Z*-score normalization and heatmap visualization of these data were performed using TBtools v2.451 (Chen et al., 2023).

### 2.10 Quantitative real-time PCR

Total RNA was extracted from the samples using the Plant RNA Extraction Mini Kit (cat. no. RNP451-02; Xinjiang Kediyuan, Shihezi, China). The *A*_260_/*A*_280_ and *A*_260_/*A*_230_ ratios of each RNA sample were measured using a Thermo NanoDrop 2000 spectrophotometer (cat. no. ND-2000; Thermo Fisher Scientific, Waltham, MA, USA) to assess the RNA purity and concentration (**Supplementary Table 8**). Subsequently, genomic DNA was removed, and the total RNA was reverse-transcribed into cDNA using a 5X All-In-One RT MasterMix kit (cat. no. G592; abm, Richmond, Canada). qRT-PCR was performed using a SYBR Green Real-time PCR Master Mix (cat. no. QPK-201; TOYOBO, Osaka, Japan) in a 20-µl reaction mixture containing 2 µl of cDNA, 10 µl of 2× SYBR Green Mix, 0.5 µl each of forward and reverse primers (10 µM), and 7 µl of ultrapure water. Gene-specific primers were designed using Primer 6 software and synthesized by Youkang Co., Ltd. (**Supplementary Table 8**). The *MsUBQ* gene was used as an internal control (housekeeping gene) for data normalization. The qRT-PCR reaction was performed on a CFX96 Real-Time PCR Detection System (cat. no. CFX Opus 96; Bio-Rad, Hercules, CA, USA) under the following thermal cycling conditions: initial denaturation at 95°C for 1 min, followed by 40 cycles of denaturation at 95°C for 10 s, annealing at 55°C for 30 s, and extension at 72°C for 30 s. Relative gene expression levels were calculated using the 2^−ΔΔ^*^C^*^t^ method.

### 2.11 Data statistics and analysis

The relative expression levels of the genes detected by qRT-PCR were calculated using the 2^−ΔΔ^*^C^*^t^ method. Statistical analysis and graphical plotting were conducted with Microsoft Excel 2024 and Origin 2021 (Li et al., 2019), respectively. All experiments were performed with three biological replicates, and the results are expressed as the mean standard deviation (SD). One-way analysis of variance (ANOVA) was performed using SPSS 26.0 software (George and Mallery, 2019). When the ANOVA yielded a significant result (*p* < 0.05), *post-hoc* multiple comparisons were conducted using the least significant difference (LSD) test to examine significant differences between each treatment group and the control group and between different treatment groups. The significance level was set at *α* = 0.05. In bar charts, bars annotated with different lowercase letters (e.g., a, b, c, or d) indicate statistically significant differences between groups (*p* < 0.05). Groups sharing the same letter are not significantly different, whereas groups marked with different letters are significantly different.

## 3 Results

### 3.1 Identification and physicochemical characterization of MsTIFY proteins

To identify *TIFY* family genes in *M. sieversii*, an HMM profile and a BLASTp search were performed. A total of 57 *MsTIFY* genes were identified and named *MsZIM1*–*MsJAZ35* according to their conserved domain and phylogenetic analysis. The length of the CDS ranged from 327 bp (*MsJAZ19*) to 2,751 bp (*MsJAZ3*). Protein lengths ranged from 109 to 917 amino acids, with the p*I* ranging from 4.52 to 10.42 and the MWs of the proteins ranging from 12.11 to 102.15 kDa. All values of the grand average of hydropathy (GRAVY) of the MsTIFY proteins were negative (**Supplementary Table 1**).

### 3.2 Conserved domain distribution and phylogenetics of the *MsTIFY* gene family

**Figure 1:**
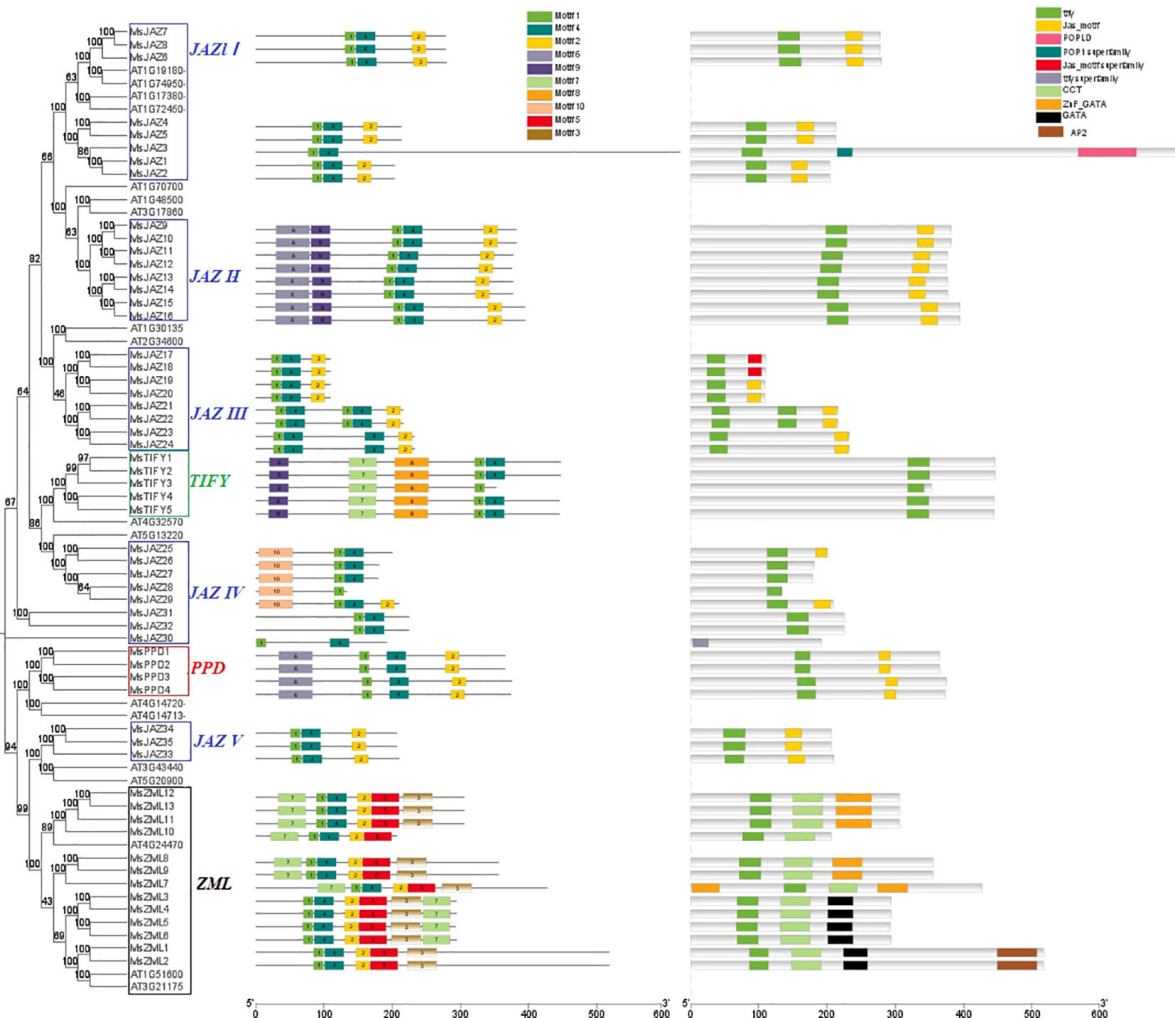
Phylogenetic tree, motif, and conserved domain analysis of the *TIFY* gene family in *Malus sieversii*. The phylogenetic tree of *M. sieversii* JAZ, PPD, and ZML proteins was constructed with the neighbor-joining (NJ) method in MEGA7. The bootstrap values were calculated with 1,000 replications for the major branches. The genes in the four subgroups are marked with different colors. The different motif compositions of *M. sieversii* TIFY, JAZ, PPD, and ZML proteins were detected using MEME. The boxes with different colors on the right denote 10 motifs. Conserved domains in the *M. sieversii* TIFY, ZML, PPD, and JAZ proteins are shown, with the different domains presented in different colors.

To investigate the classification and evolutionary relationships of the *MsTIFY* gene family, the protein sequences of 18 *AtTIFY* and 57 *MsTIFY* genes were used to construct a neighbor-joining phylogenetic tree. As shown in **Figure 1**, the 57 *MsTIFY* members were categorized into four subfamilies: *JAZ* (35 members), *ZML* (13 members), *TIFY* (five members), and *PPD* (four members). The JAZ subfamily was further subdivided into five subgroups (JAZ I–V), with JAZ V containing the fewest members (*n* = 3) and JAZ I–IV, each containing eight members. There were 10 motifs identified, named motif 1 to motif 10. Motif 1 (TIFY motif) was distributed in all 57 *MsTIFY* members. Genes belonging to the same phylogenetic group were found to have similar motif structures. Domain architecture analysis revealed subfamily-specific features: the TIFY subfamily contains the TIFY domain; the JAZ subfamily contains the TIFY domain and the C-terminal Jas domain; the PPD subfamily contains the TIFY domain, the N-terminal PPD domain, and a Jas domain lacking the PY motif; and the ZML subfamily contains the TIFY domain, the CCT motif, and a GATA zinc finger domain (**Figure 1**).

### 3.3 Subcellular localization prediction and secondary structure analysis

Subcellular localization prediction showed that the majority of the members of the *MsTIFY* gene family are localized in the nucleus, while a small number of family members are localized in the chloroplast, cytoplasm, mitochondria, and plasma membrane. Secondary structure analysis revealed that all MsTIFY proteins contain α-helices, β-turns, random coils, and extended strands. Among these, random coils and α-helices constitute the predominant secondary structure types within this family. The composition ranges were 30.73%–75.70% for random coils, 9.87%–40.10% for α-helices, 6.57%–22.24% for extended strands, and 1.77%–9.38% for β-turns (**Supplementary Table 2**).

### 3.4 Comprehensive promoter analysis and regulatory element characterization

**Figure 2:**
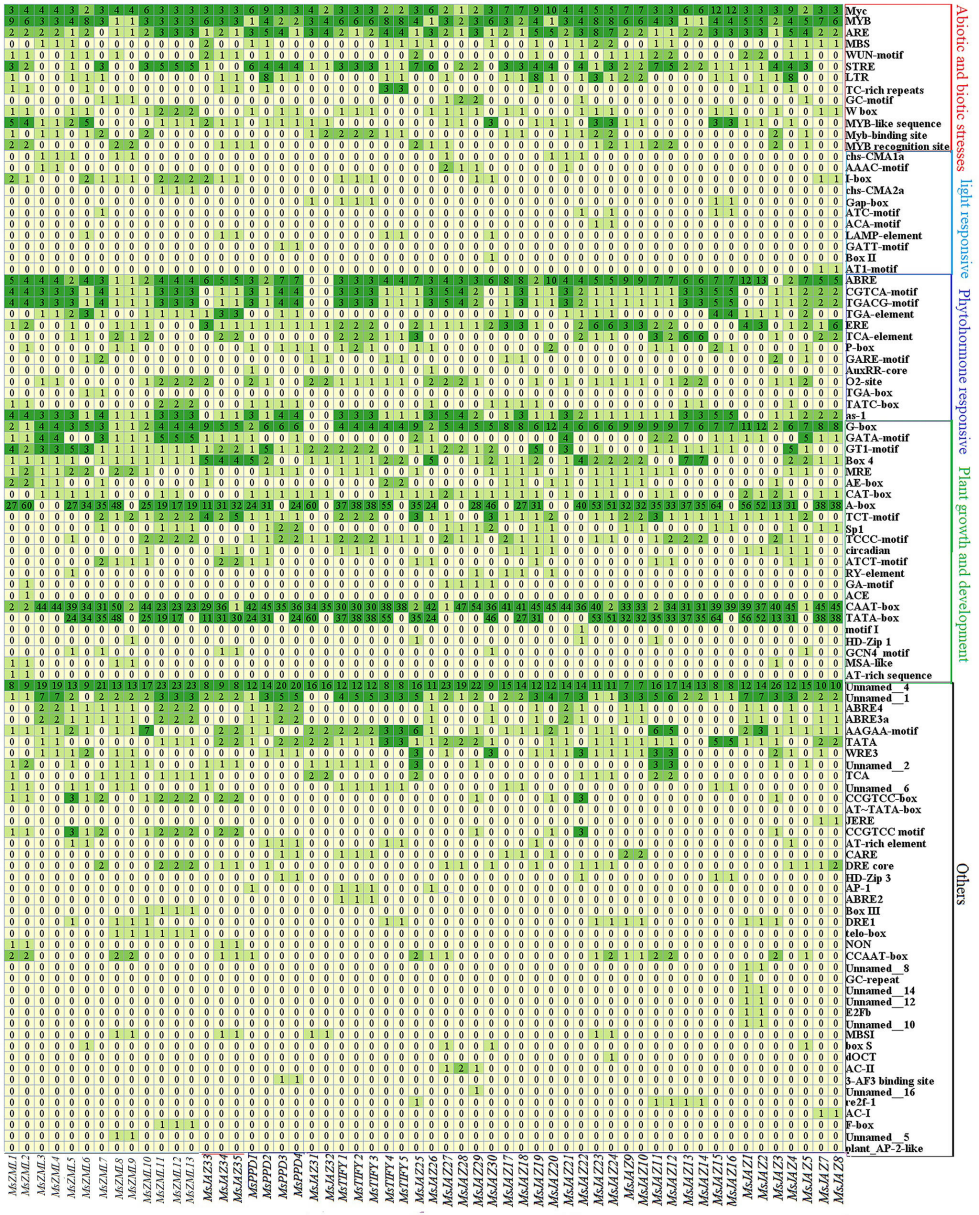
The number of different *cis*-acting elements in the 2-kb upstream promoter regions of *MsTIFY* genes. The promoter elements identified in the 57 *MsTIFY* genes were classified into four categories: stress response elements, light-responsive elements, hormone-responsive elements, and elements associated with plant growth and development, in addition to other elements. The histogram shows the distribution of the number of *cis*-acting elements.

Analysis of the 2-kb upstream promoter regions of the 57 *MsTIFY* genes identified 103 distinct *cis*-acting regulatory elements (**Figure 2**). Core promoter elements essential for RNA polymerase II binding and transcription initiation, specifically the CAAT box and TATA box, were detected. In addition, several stress-responsive elements were found, including LTR (involved in low-temperature responsiveness), MBS (associated with drought inducibility), and TC-rich repeats (related to defense and stress responses). *Cis*-acting elements involved in phytohormone responses are prevalent among the majority of the family members. These include the ABRE (abscisic acid responsiveness), the TGACG and CGTCA motifs (both involved in MeJA responsiveness), and elements associated with auxin (TGA element, O2 site, AuxRR core, and TGA box), gibberellin (TATC box, P box, and GARE motif), and salicylic acid (TCA element) signaling. Elements linked to anaerobic or hypoxic conditions, such as the ARE and the CCAAT boxes, were also present. Several elements involved in developmental and tissue-specific regulation were identified, included CAT-box (meristem expression), RY-element (seed-specific regulation), GCN4_motif (endosperm expression), HD-Zip 1 (involved in palisade mesophyll cell differentiation), MSA-like (cell cycle regulation), and AT-rich sequence (root-specific regulation).

### 3.5 Chromosomal localization and intraspecies synteny analysis reveal gene duplication patterns

**Figure 3:**
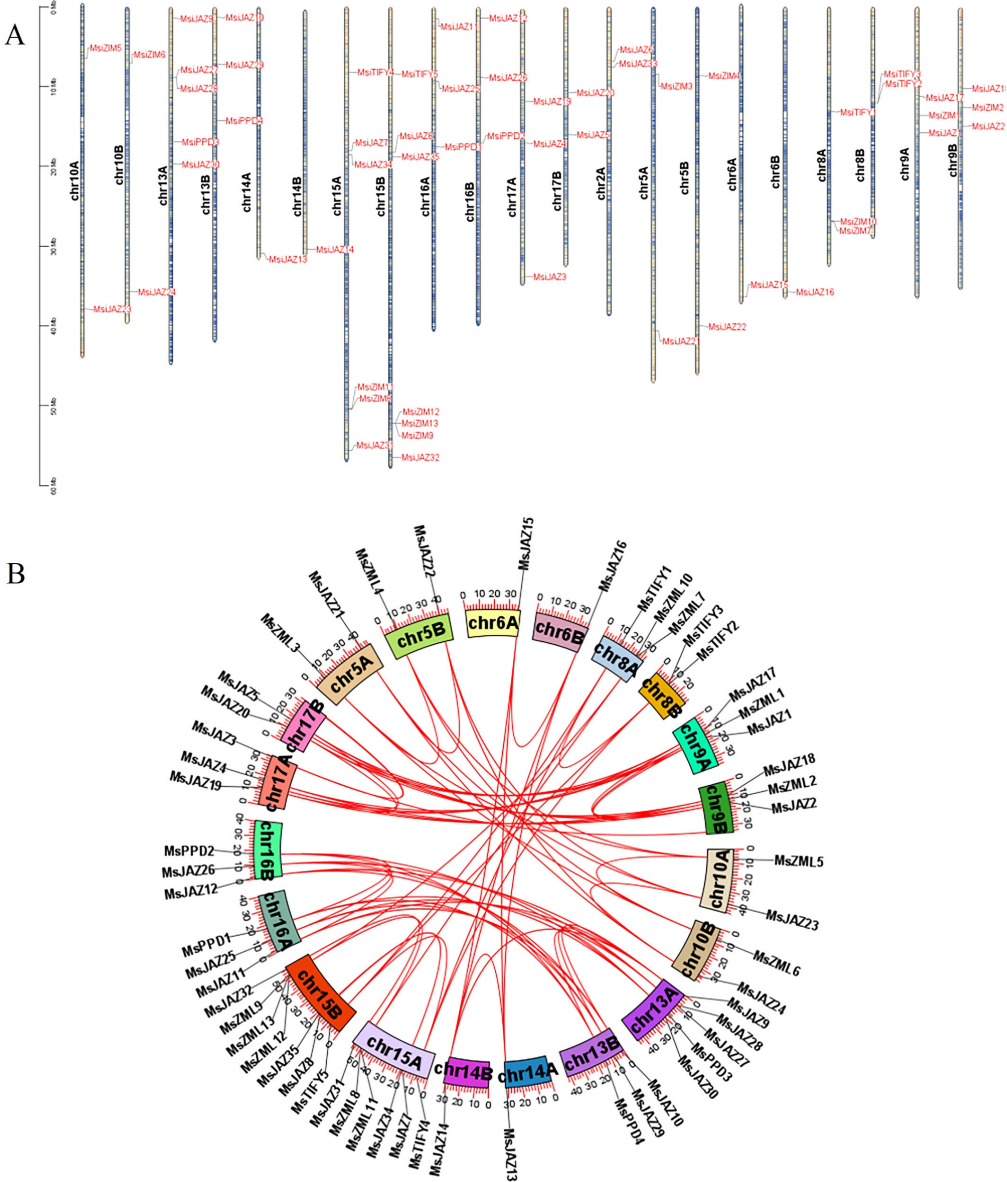
Chromosomal localization and intraspecific synteny analysis. **(A)** Distribution of the *MsTIFY* genes on chromosomes. **(B)** Analysis of the intraspecific syntenic relationships between *MsTIFY* genes.

Chromosomal localization analysis of the *MsTIFY* gene family members revealed that 21 chromosomes of *M. sieversii* contain an unequal distribution of 57 *MsTIFY* genes. Chr15B contained the largest number of *MsTIFY* genes (*n* = 7) (**Figure 3A**), while chr6A and chr6B contained the fewest (*n* = 1). Tandemly duplicated gene sets include (Ms*JAZ27*, Ms*JAZ28*), (*MsZIM11*, *MsZIM8*), (*MsZIM9*, *MsZIM12*, *MsZIM13*), (*MsZIM10*, *MsZIM7*), and (*MsTIFY3*, *MsTIFY2*). Intraspecies collinearity analysis within the *M. sieversii* genome identified 69 segmentally duplicated gene pairs of *MsTIFY* (**Figure 3B**). In the ZML subfamily, a total of 13 collinear gene pairs were identified, including (*MsZML1*, *MsZML2*), (*MsZML3*, *MsZML4*), (*MsZML5*, *MsZML6*), (*MsZML5*, *MsZML3*), (*MsZML5*, *MsZML4*), and (*MsZML6*, *MsZML3*), among others. In the PPD subfamily, a total of six collinear gene pairs were detected, namely, (*MsPPD1*, *MsPPD2*), (*MsPPD3*, *MsPPD4*), (*MsPPD3*, *MsPPD1*), (*MsPPD3*, *MsPPD2*), (*MsPPD4*, *MsPPD1*), and (*MsPPD4*, *MsPPD2*). In the TIFY subfamily, a total of six collinear gene pairs were found: (*MsTIFY1*, *MsTIFY3*), (*MsTIFY4*, *MsTIFY5*), (*MsTIFY4*, *MsTIFY1*), (*MsTIFY4*, *MsTIFY3*), (*MsTIFY5*, *MsTIFY1*), and (*MsTIFY5*, *MsTIFY3*). In the JAZ subfamily, a total of 44 collinear gene pairs were observed, including (*MsJAZ34*, *MsJAZ35*), (*MsJAZ34*, *MsJAZ33*), (*MsJAZ35*, *MsJAZ33*), (*MsJAZ31*, *MsJAZ32*), and (*MsJAZ25*, *MsJAZ26*), among others (**Supplementary Table 3**).

The collinear genes on these chromosomes are located in close proximity and are arranged in the same linear order. *K*_a_/*K*_s_ analysis of the 59 *TIFY* syntenic gene pairs in the *M. sieversii* genome revealed that the majority of gene pairs (*K*_a_/*K*_s_ < 1) are primarily under purifying selection, indicating functional conservation. In contrast, one pair (e.g., *MsJAZ11* and *MsJAZ12*) exhibited a *K*_a_/*K*_s_ ratio of 1.43 (>1), suggesting that this gene pair may have experienced positive selection after speciation, implying adaptive functional divergence (**Supplementary Table 9**).

### 3.6 Comprehensive collinearity analysis of *MsTIFY* and *TIFY* genes from different plant species

**Figure 4:**
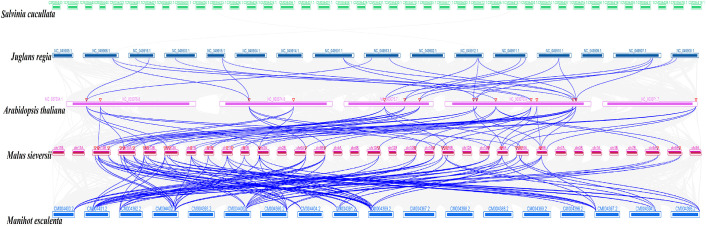
Comparative synteny analysis of the *TIFY* genes between *Malus sieversii* and four plant species: *Salvinia cucullata*, *Juglans regia*, *Arabidopsis thaliana*, and *Manihot esculenta*. The syntenic relationships of the *TIFY* genes between *M. sieversii* and each of the four species are displayed in the figure. This analysis reveals the homology and evolutionary conservation of the *TIFY* genes among these species.

A comprehensive collinearity analysis of *TIFY* genes was conducted across five plant species: *M. sieversii*, *S. cucullata*, *J. regia*, *A. thaliana*, and *M. esculenta* (**Figure 4**). The results revealed 52 *TIFY* gene-related collinear gene pairs between *M. sieversii* and *A. thaliana*. Specifically, the gene *NM_102753.4* corresponds to the collinear genes *MsJAZ21*, *MsJAZ22*, *MsJAZ23*, and *MsJAZ24*; *NM_001334477.1* corresponds to *MsJAZ9*, *MsJAZ10*, *MsJAZ11*, and *MsJAZ12*; *NM_117555.4* corresponds to *MsPPD1*, *MsPPD2*, *MsPPD3*, and *MsPPD4*; and *NM_105904.4* corresponds to *MsJAZ1*, *MsJAZ2*, *MsJAZ4*, *MsJAZ5*, *MsJAZ6*, *MsJAZ7*, and *MsJAZ8*, among others (**Supplementary Table 4**). Between *M. sieversii* and *J. regia*, a total of 93 *TIFY* gene-related collinear gene pairs were identified. For instance, *XM_018959192.2* corresponds to *MsJAZ7*, *MsJAZ8*, *MsJAZ4*, *MsJAZ5*, *MsJAZ6*, *MsJAZ1*, and *MsJAZ2*; *XM_018954845.2* corresponds to *MsJAZ23*, *MsJAZ24*, *MsJAZ21*, and *MsJAZ22*; *XM_018964148.2* corresponds to *MsJAZ23*, *MsJAZ24*, *MsJAZ21*, and *MsJAZ22*; and *XM_018963069.2* corresponds to *MsPPD3*, *MsPPD4*, *MsPPD1*, and *MsPPD2*, among others (**Supplementary Table 4**). Between *M. sieversii* and *M. esculenta*, 115 *TIFY* gene-related collinear gene pairs were detected. Examples include *Manes.03G042500.1*, which corresponds to *MsJAZ7*, *MsJAZ34*, *MsJAZ8*, *MsJAZ35*, *MsJAZ4*, *MsJAZ5*, *MsJAZ6*, *MsJAZ1*, and *MsJAZ2*; *Manes.03G065500.2*, which corresponds to *MsTIFY4*, *MsTIFY5*, *MsTIFY1*, and *MsTIFY3*; *Manes.03G055200.1*, which corresponds to *MsJAZ7*, *MsJAZ34*, *MsJAZ35*, and *MsJAZ33*; and *Manes.04G147900.1*, which corresponds to *MsJAZ7*, *MsJAZ8*, *MsJAZ4*, *MsJAZ5*, *MsJAZ6*, *MsJAZ1*, and *MsJAZ2*, among others (**Supplementary Table 4**). No *TIFY* gene-related collinear gene pairs were identified between *M. sieversii* and *S. cucullata*.

*K*_a_/*K*_s_ analysis was performed on syntenic *TIFY* gene pairs among species. The mean *K*_a_/*K*_s_ ratios were 0.23 for pairs between *M. sieversii* and *A. thaliana*, 0.26 for those with *J. regia*, and 0.28 for those with *M. esculenta*. All inter-species gene pairs exhibited *K*_a_/*K*_s_ ratios less than 1 (ranging from 0.06 to 0.48), indicating that the *TIFY* gene family has been subject to generally strong purifying selection constraints throughout its evolutionary history. The *K*_a_, *K*_s_, and *K*_a_/*K*_s_ values for each syntenic gene pair are detailed in **Supplementary Table 9**.

### 3.7 GO functional annotation prediction of *MsTIFY* genes

**Figure 5:**
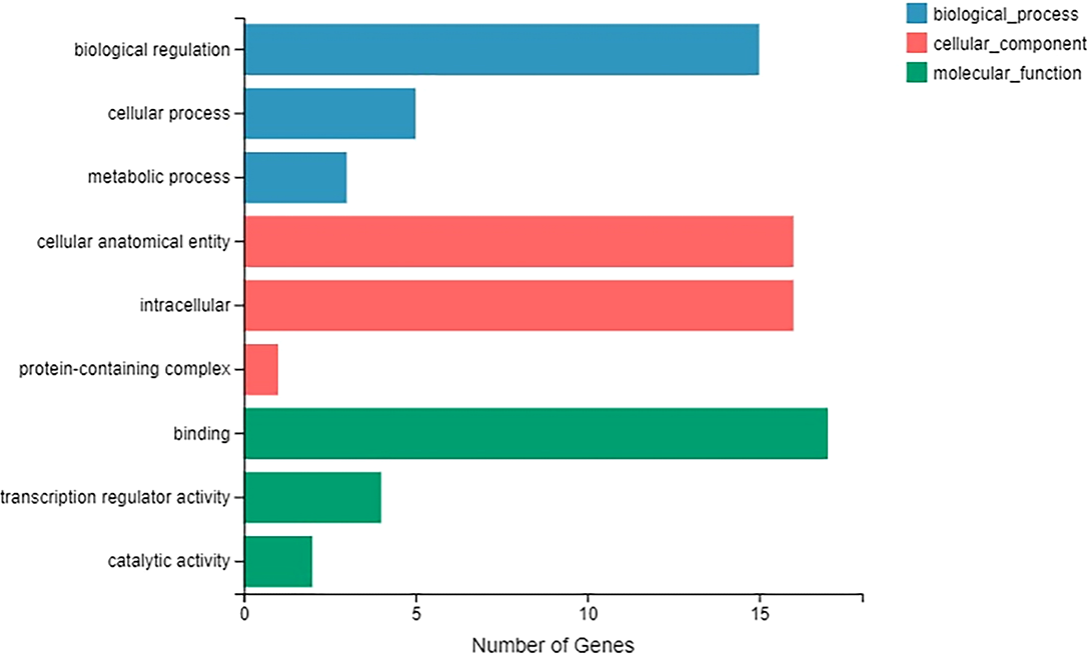
Statistical chart of the Gene Ontology (GO) terms annotated for the *MsTIFY* gene members in *Malus sieversii*. A total of 18 *MsTIFY* genes were annotated. The annotated terms are listed as follows: biological process: biological regulation, cellular process, and metabolic process; cellular component: cellular anatomical entity, intracellular, and protein-containing complex; and molecular function: binding, transcription regulator activity, and catalytic activity.

The functional annotation prediction of *MsTIFY* genes was performed using Blast2GO, with 18 *MsTIFY* genes annotated (**Figure 5**). Within the biological process category, 15 genes were assigned to “biological regulation,” five genes were associated with “cellular process,” and three genes were linked to “metabolic process.” Within the cellular component category, 16 genes were assigned to “cellular anatomical entity” and “intracellular,” and one gene was assigned to “protein-containing complex.” Within the molecular function category, 17 genes were assigned to “binding,” four genes were annotated with “transcription regulator activity,” and two genes were assigned to “catalytic activity” (**Supplementary Table 5**).

### 3.8 Expression pattern analysis of the *MsTIFY* gene family in *Malus sieversii*

**Figure 6:**
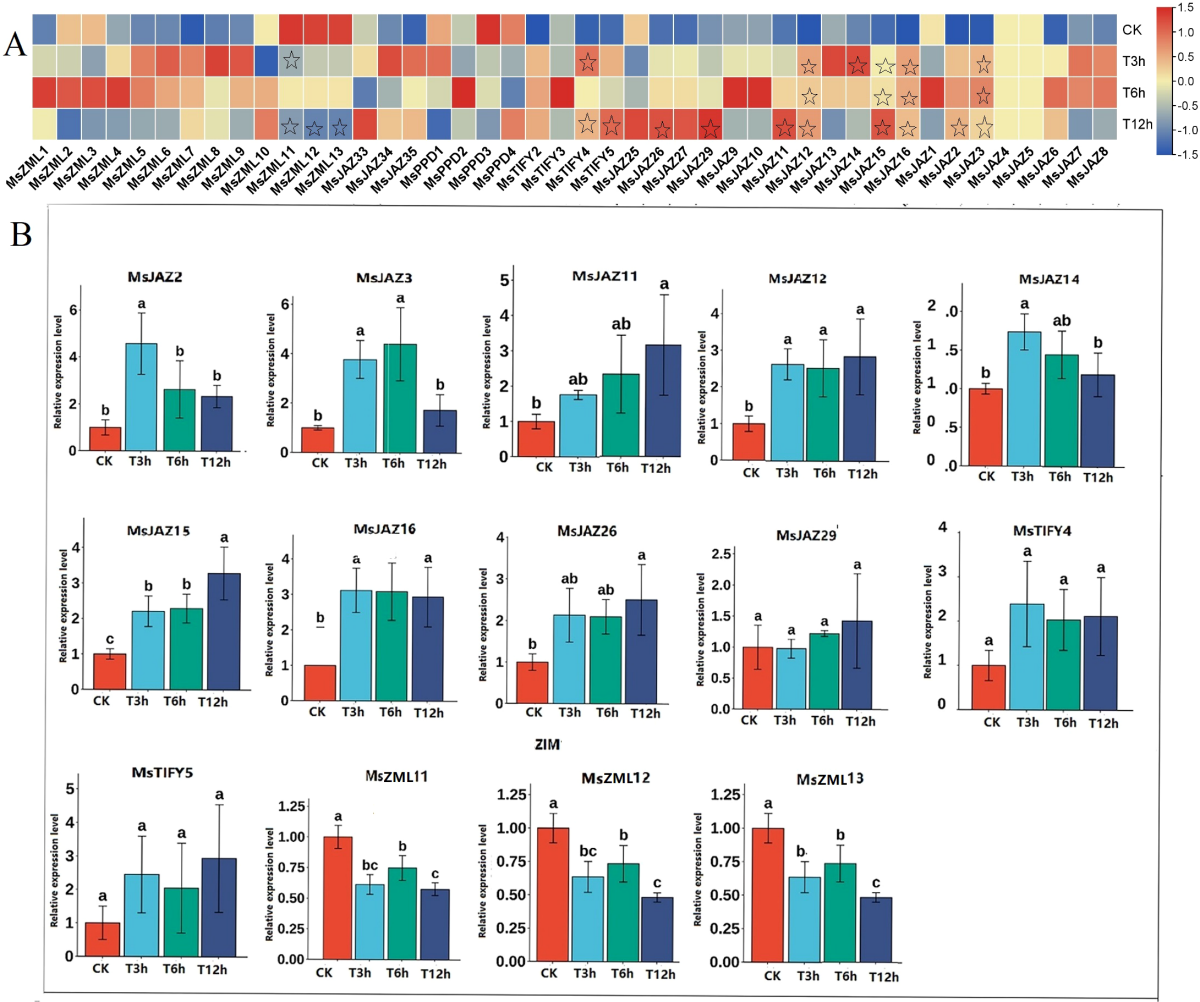
Expression profiles of *MsTIFY* genes under cold stress. **(A)** Expression levels of *MsTIFY* genes under cold stress. The experiment consisted of three groups: a control group (*CK*; with three biological replicates designated CK1, CK2, and CK3); a cold stress treatment group for 3 h (*T3h*; with three replicates T3h1, T3h2, and T3h3); a cold stress treatment group for 6 h (*T6h*; with three replicates T6h1, T6h2, and T6h3); and a cold stress treatment group for 12 h (*T12h*; with three replicates T12h1, T12h2, and T12h3). Based on the mean FPKM (fragments per kilobase of transcript per million mapped reads) values of each gene across treatments, *Z*-score normalization of the expression data and heatmap visualization were performed using TBtools v2.451, in which *blue* and *red* represent downregulated and upregulated expression, respectively. Differentially expressed genes (DEGs) were screened using the following criteria: |fold change| ≥ 1.5 or |fold change| ≤ 0.5 and an adjusted *p*-value (*p*_adj_) <0.05. In the heatmap, genes showing significant differential expression relative to the control group are marked with an *asterisk*. **(B)** Quantitative real-time PCR (qRT-PCR) validation of *MsJAZ2*, *MsJAZ3*, *MsJAZ11*, *MsJAZ12*, *MsJAZ14*, *MsJAZ15*, *MsJAZ16*, *MsJAZ26*, *MsJAZ29*, *MsTIFY4*, *MsTIFY5*, *MsZML11*, *MsZML12*, and *MsZML13* under low-temperature stress, with *MsUBQ* serving as the internal control. Data are presented as the mean ± SD (*n* = 3). *Different lowercase letters* (*a*–*d*) indicate significant differences between groups (*p* < 0.05, one-way ANOVA followed by a LSD *post-hoc* test), while the *same letter* indicates no significant difference.

Based on transcriptome sequencing data, this study systematically analyzed the expression patterns of the *MsTIFY* gene family in the leaves of *M. sieversii* under cold stress (**Figure 6A**). The expression levels of multiple members of this family changed significantly under cold stress. *MsTIFY4*, *MsTIFY5*, *MsJAZ2*, *MsJAZ3*, *MsJAZ11*, *MsJAZ12*, *MsJAZ14*, *MsJAZ15*, *MsJAZ16*, *MsJAZ26*, and *MsJAZ29* were significantly upregulated under cold stress, while *MsZML11*, *MsZML12*, and *MsZML13* were significantly downregulated (**Supplementary Table 6**). The expression levels of selected *MsTIFY* genes were further analyzed using qRT-PCR, and the trends were largely consistent with the transcriptome analysis results (**Figure 6B**).

**Figure 7:**
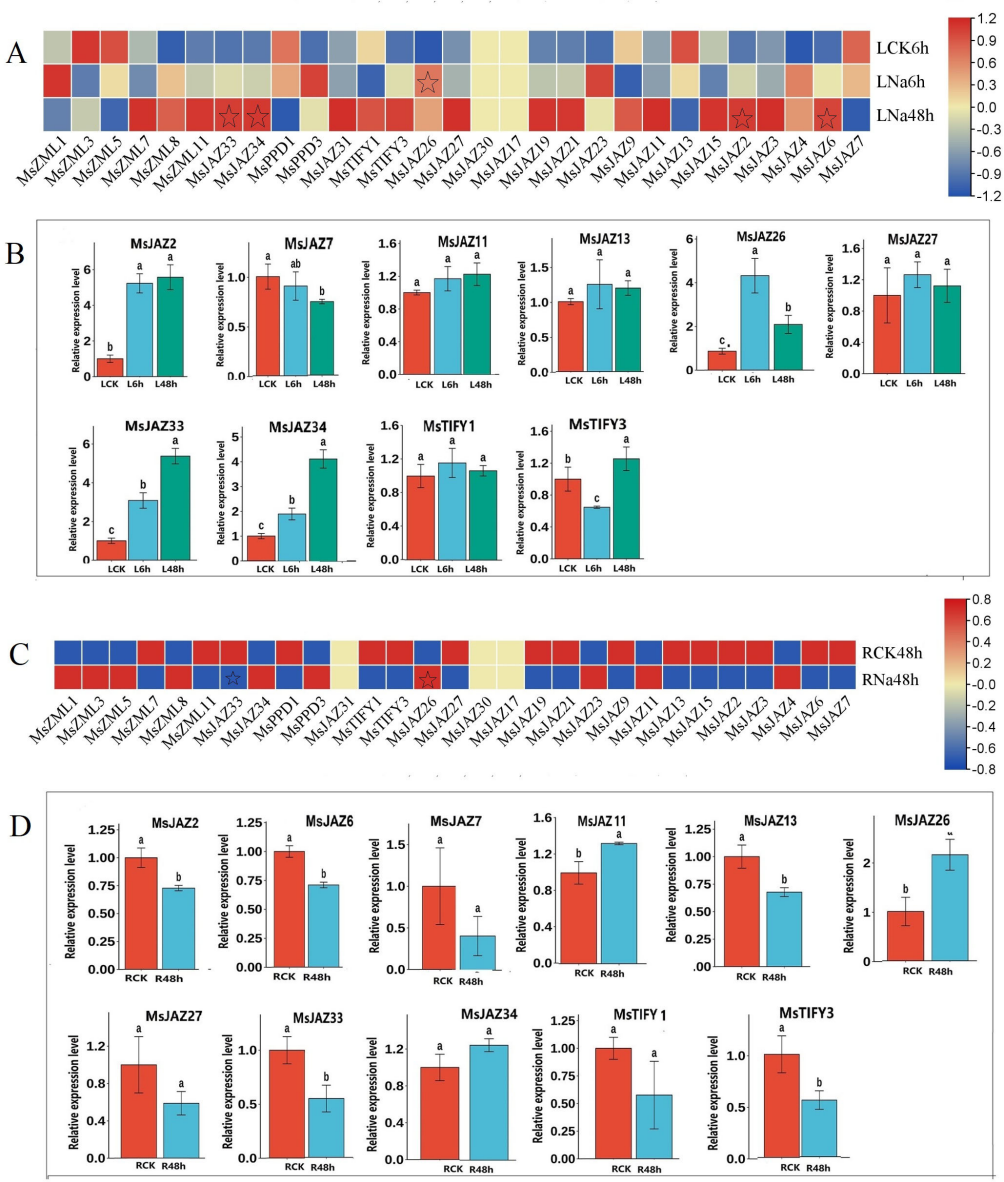
Expression profiles of *MsTIFY* genes under salt stress. **(A, C)** The experiments consisted of three groups for leaves: a control group (leaf samples designated *LCK*, with three biological replicates LCK1, LCK2, and LCK3), a salt treatment group for 6 h (leaf samples designated *L6h*, with three replicates L6h1, L6h2, and L6h3), and a salt treatment group for 48 h (leaf samples designated *L48h*, with three replicates L48h1, L48h2, and L48h3) **(A)**; and two groups for roots: a control group (root samples designated *RCK*, with three biological replicates RCK1, RCK2, and RCK3) and a salt treatment group for 48 h (root samples designated *R48h*, with three replicates R48h1, R48h2, and R48h3) **(C)**. Based on the mean FPKM (fragments per kilobase of transcript per million mapped reads) values of each gene across treatments, *Z*-score normalization of the expression data and heatmap visualization were performed using TBtools v2.451, in which *blue* and *red* represent downregulated and upregulated expression, respectively. Differentially expressed genes (DEGs) were screened using the following criteria: |fold change| ≥ 1.5 or |fold change| ≤ 0.5 and an adjusted *p*-value (*p*_adj_) <0.05. In the heatmap, genes showing significant differential expression relative to the control group are marked with an *asterisk*. **(B, D)** Quantitative real-time PCR (qRT-PCR) validation of *MsJAZ2*, *MsJAZ7*, *MsJAZ11*, *MsJAZ13*, *MsJAZ26*, *MsJAZ27*, *MsJAZ33*, *MsJAZ34*, *MsTIFY1*, and *MsTIFY3* in leaves **(B)** and in roots **(D)** under salt stress. In both, *MsUBQ* served as the internal control. Data are presented as the mean ± SD (*n* = 3). *Different lowercase letters* (*a*–*d*) indicate significant differences between groups (*p* < 0.05, one-way ANOVA followed by a LSD *post-hoc* test), while the *same letter* indicates no significant difference.

Under salt stress conditions (**Supplementary Table 6**), the *MsTIFY* genes that showed significant upregulation in the leaf tissue of *M. sieversii* included *MsJAZ33*, *MsJAZ34*, *MsJAZ26*, *MsJAZ2*, and *MsJAZ6* (**Figure 7A**). In the root tissue of *M. sieversii*, the expression of *MsJAZ26* was significantly upregulated, while that of *MsJAZ33* was markedly downregulated (**Figure 7C**). qRT-PCR analysis of selected *MsTIFY* genes revealed expression trends largely consistent with the transcriptome data (**Figures 7B–D**).

**Figure 8:**
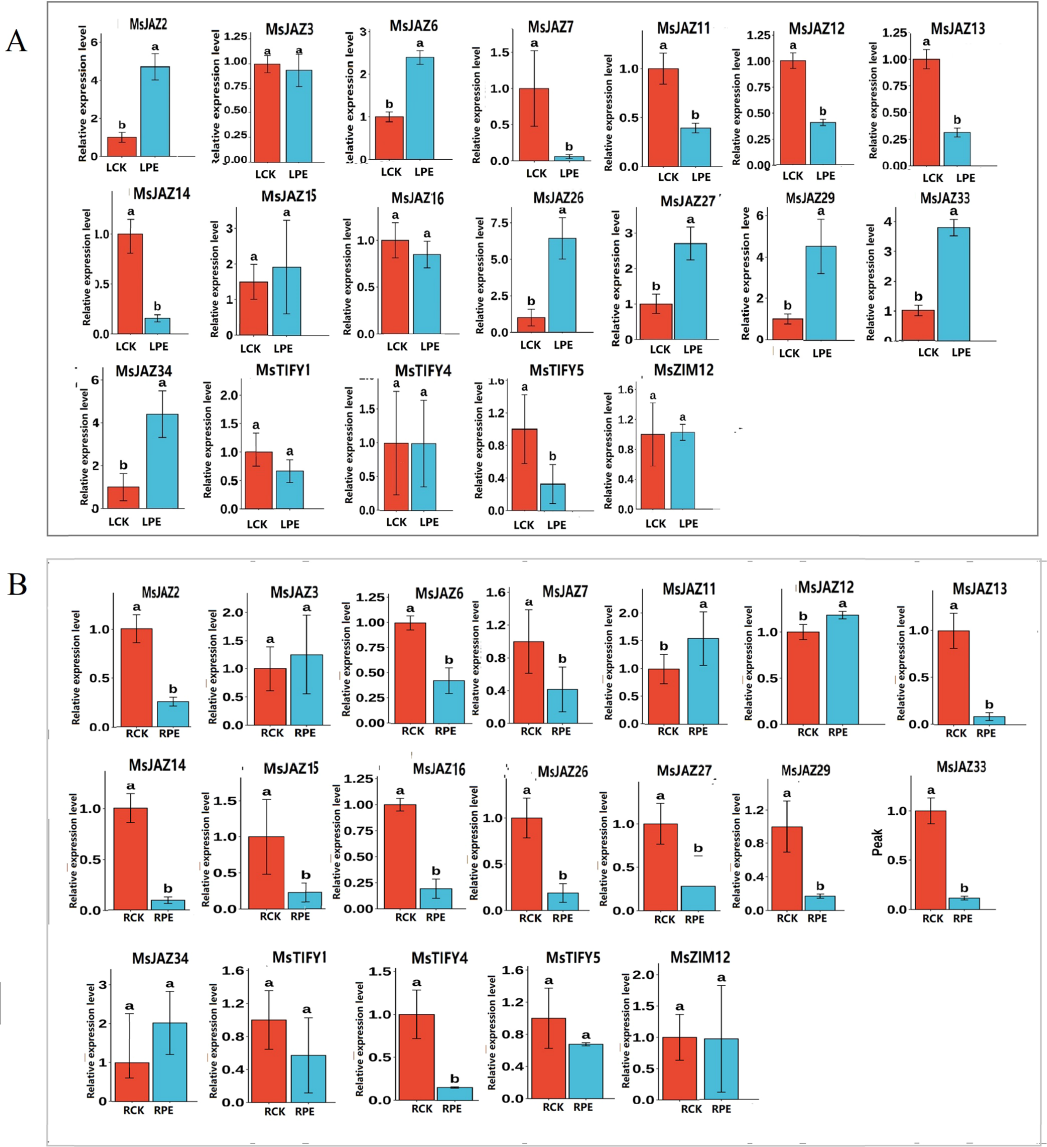
Expression profiles of *MsTIFY* genes under drought stress. **(A, B)** Quantitative real-time PCR (qRT-PCR) validation of *MsJAZ2*, *MsJAZ3*, *MsJAZ6*, *MsJAZ7*, *MsJAZ11*, *MsJAZ12*, *MsJAZ13*, *MsJAZ14*, *MsJAZ15*, *MsJAZ16*, *MsJAZ26*, *MsJAZ27*, *MsJAZ29*, *MsJAZ33*, *MsJAZ34*, *MsTIFY1*, *MsTIFY4*, *MsTIFY5*, and *MsZML12* in leaves **(A)** and roots **(B)** under drought stress. In both, *MsUBQ* served as the internal control. Error bars represent the standard deviation (SD) of three biological replicates. Data are presented as the mean ± SD (*n* = 3). Different lowercase letters (a–d) indicate significant differences between groups (*p* < 0.05, one-way ANOVA followed by a LSD *post-hoc* test), while the same letter indicates no significant difference.

*MsTIFY* genes that exhibited significant responses to low-temperature and salt stress were examined via qRT-PCR in the leaves and roots of *M. sieversii* under drought stress. In the leaves, the expression of *MsJAZ33*, *MsJAZ34*, *MsJAZ26*, *MsJAZ27*, *MsJAZ15*, *MsJAZ2*, and *MsJAZ6* was upregulated, while that of *MsTIFY1*, *MsJAZ11*, and *MsJAZ12* was downregulated (**Figure 8A**). In the roots, *MsJAZ11* and *MsJAZ12* showed increased expression, whereas the expression of *MsJAZ33*, *MsJAZ34*, *MsTIFY1*, *MsTIFY4*, *MsJAZ26*, *MsJAZ27*, *MsJAZ15*, *MsJAZ16*, *MsJAZ2*, and *MsJAZ6* was reduced (**Figure 8B**).

### 3.9 Subcellular localization of *MsJAZ26* and *MsJAZ2*

**Figure 9:**
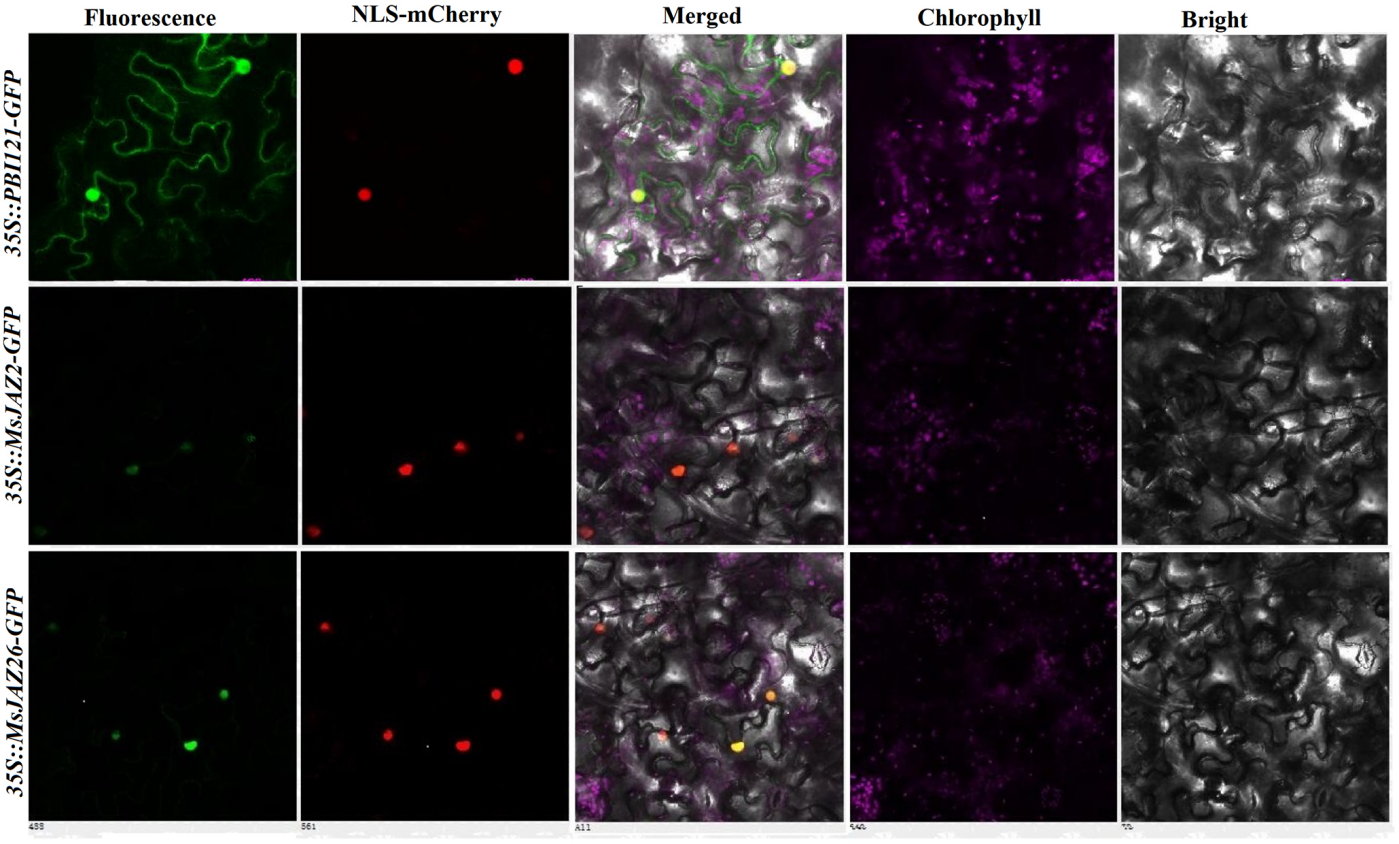
Subcellular localization results of *MsJAZ26* and *MsJAZ2.* The *pBI121–MsJAZ2–GFP* and *pBI121–MsJAZ26–GFP* fusion expression vectors were constructed and individually co-transformed with the nuclear marker *AtHY5-CHERRY* into GV3101 competent cells. The resulting GV3101 strains were mixed at a 1:1 ratio and infiltrated into *Nicotiana benthamiana* leaves. After dark incubation for 24–36 h, confocal laser scanning microscopy revealed that the green fluorescent protein (GFP) fluorescence signals of *MsJAZ2* and *MsJAZ26* co-localized with the red fluorescence of the nuclear marker in the nuclear region, indicating that both *MsJAZ2* and *MsJAZ26* are localized in the nucleus.

To further determine their subcellular localization, the expression vectors *35S:MsJAZ2-GFP* and *35S:MsJAZ26-GFP* were constructed and transiently expressed in *N. benthamiana* leaves. Using laser scanning confocal microscopy, the GFP fusion proteins were excited at 488 nm, and the target protein fluorescence signals appeared green. The nuclear marker protein mCherry was excited at 561 nm, and its fluorescence signal appeared red. The results showed that the fluorescent signals of the *35S:MsJAZ2-GFP* and *35S:MsJAZ26-GFP* fusion proteins co-localized with the nuclear marker *AtHY5-mCherry* specifically in the nuclear region, indicating that *MsJAZ2* and *MsJAZ26* are primarily localized in the nucleus (**Figure 9**).

## 4 Discussion

The *TIFY* gene family is a plant-specific family of transcription factors that regulates plant development, physiological processes, and stress responses. To date, the most extensively characterized subfamily is the JAZ subfamily (Yan et al., 2009; Ye et al., 2009; Demianski et al., 2012). Elucidating the molecular mechanisms of the *TIFY* family will help clarify the regulatory networks underlying plant stress tolerance and provide a theoretical foundation for the breeding of new cultivars with enhanced adaptability. However, little is known about the expression and function of the *MsTIFY* genes in *M. sieversii*—the wild ancestor of the modern cultivated apple whose germplasm harbors important agronomic traits (Volk et al., 2005; Yan et al., 2008). Therefore, based on the published whole-genome sequencing data of *M. sieversii* (Sun et al., 2020), this study conducted a genome-wide survey of the *TIFY* genes in *M. sieversii* and explored their evolutionary history and expression diversity under multiple stress conditions.

### 4.1 Gene expansion driven by whole-genome duplication and tandem duplication

A total of 57 *MsTIFY* genes were identified in the genome of *M. sieversii*, a number significantly higher than that reported in rice, poplar, maize, and other species. The expansion of this gene family can be attributed to two synergistic mechanisms: whole-genome duplication (WGD) and tandem duplication. WGD and tandem duplication are recognized as major driving forces of gene family expansion (Zhang, 2003; Ganko et al., 2007; Jiao et al., 2011; Wang et al., 2012; Mable et al., 2018). WGD can globally double the copy number of paralogous genes in a single event (Johri et al., 2022), and recent studies have indicated that the Rosaceae family experienced a WGD event (Zhang et al., 2025). This, combined with active tandem duplication and species-specific gene retention mechanisms in apple (Lallemand et al., 2023), has collectively resulted in an increase in gene copy number.

### 4.2 Evolutionary conservation and adaptive positive selection in the *TIFY* gene family

*K*_a_/*K*_s_ analysis of intraspecific and interspecific syntenic *TIFY* gene pairs in *M. sieversii* revealed that the majority of *TIFY* genes are c (**Supplementary Table 9**). This is consistent with the results of *K*_a_/*K*_s_ analyses of the *TIFY* gene family in multiple species (Heidari et al., 2021; Li et al., 2024a), indicating that the *TIFY* family has maintained relatively conserved biological functions during evolution—for instance, serving as key regulators of the JA signaling pathway and participating in plant growth, development, and defense responses (Howe et al., 2019a, b). Notably, the *MsJAZ11*–*MsJAZ12* gene pair in the *M. sieversii* genome exhibited *K*_a_/*K*_s_ > 1, implying that positive selection may have driven the adaptive functional divergence of this gene pair following speciation. Previous studies have detected signals of positive selection in *JAZ* genes from species such as maize and the tea plant, which may be associated with adaptation to specific environmental stresses (Han and Luthe, 2021; Xiong et al., 2025). Given that *M. sieversii* is a Tertiary relict plant distributed in the arid mountainous regions of Central Asia and that its population genetic variation is significantly influenced by environmental factors such as annual temperature range and temperature seasonality (Volk et al., 2005; Yan et al., 2008; Zhang et al., 2022), the positively selected *JAZ* gene pair may have conferred local adaptive advantages under specific ecological conditions.

### 4.3 Molecular mechanisms underlying the emergence of diversity

The *TIFY* genes of *M. sieversii* exhibit extensive diversity in both physicochemical properties and domain architectures, a diversity that originates from multiple evolutionary driving forces at the genomic level (Eirín-López et al., 2012). First, gene duplication serves as the core mechanism of family expansion: whole-genome, segmental, and tandem duplications occur frequently (Cannon et al., 2004; Rensing, 2018), generating abundant homologous copies. Subsequently, point mutations, insertions, or deletions alter the amino acid length and cause p*I* shifts in the encoded proteins (Studer et al., 2013). These sequence divergences become fixed in populations, providing the material basis for functional differentiation. Second, alternative splicing generates diverse mRNA splice isoforms through the selective use of exons, which are then translated into protein isoforms that differ in domain composition—for example, containing or lacking a particular domain (Breitbart et al., 1987). A classic example is the *Arabidopsis* JAZ10 splice variant JAZ10.4, which retains the TIFY domain but lacks the C-terminal Jas domain. Consequently, it escapes COI1-mediated recognition and degradation and forms non-functional heterodimers with other JAZ proteins via the TIFY domain, thereby exerting a dominant-negative regulatory effect. This mechanism greatly expands the repertoire of domain combinations and functional outputs without altering gene copy number (Chung and Howe, 2009). Third, domain shuffling driven by exon recombination can cause members of the same gene family to diverge into distinct subgroups (Nouaud et al., 2003). These domain rearrangements remodel the protein interaction surfaces, steering different subfamilies onto entirely distinct functional trajectories.

### 4.4 Functional diversification arising from *TIFY* diversity

The diversity in the physicochemical properties and domain architectures directly shapes the functional division of labor among *TIFY* family members across different signaling pathways and biological processes (Yang et al., 2020). First, regarding protein stability and hormone response sensitivity: the majority of TIFY proteins are predicted to be unstable, which is consistent with their role as repressors that require rapid turnovers (Howe et al., 2018). In contrast, stable variants lacking the Jas domain can constitutively suppress signaling (Chung and Howe, 2009). This “stability dimorphism” allows plants to mount rapid, reversible responses to jasmonate (JA) signals or to impose long-term feedback inhibition. Second, the specificity of protein–protein interaction networks and signal bifurcation: minor variations in the Jas domain determine hormone perception specificity. Sequence differences in the Jas domain of different JAZ members affect their affinity for the COI1 receptor and can even discriminate between different JA bioactive molecules, such as JA-Ile and coronatine (Melotto et al., 2008). The TIFY core motif (TIF[F/Y]XG) mediates homo- and heterodimerization (Chung and Howe, 2009), enabling plants to assemble specific repressor complexes targeting distinct downstream transcription factors such as MYC, EIN3, and RGA. Thereby, it achieves crosstalk between JA and the signaling pathways of auxin, ethylene, gibberellin, and others (Zhu et al., 2011; Yang et al., 2012; Chini et al., 2009). Third, subcellular compartmentalization and environmental adaptation: the wide distribution of isoelectric points, ranging from strongly acidic to strongly basic, endows different TIFY proteins with distinct charge states in the specific pH microenvironments of the nucleus and cytoplasm, influencing nonspecific interactions with nucleic acids or proteins (Kurotani et al., 2019; Zhang et al., 2025). Fourth, functional divergence at the subfamily level is evident. The *JAZ* subfamily, via its TIFY and Jas domains, specifically functions as the core repressor of jasmonate (JA) signaling and serves as a central hub for plant defense and development (Howe and Yoshida, 2019a, b). The *ZML* subfamily, which possesses GATA zinc finger and CCT domains, likely directly binds DNA to regulate gene expression and participates in the control of photoperiod and flowering time, acting independently of JA signaling (Shikata et al., 2004). The *PPD* subfamily is characterized by a distinctive PPD domain that enables its involvement in leaf morphogenesis, fruit development, and organ size control (Li and Zhu, 2020). The *TIFY* subfamily, which contains only the TIFY domain, remains functionally elusive and may serve as a simple antagonistic module involved in modulating protein–protein interaction networks (Thireault et al., 2015). Finally, diversified responses through phosphorylation regulation (Howe et al., 2018): because different TIFY proteins differ in the number and positions of phosphorylation sites, they are recognized by specific kinases. For instance, under stress conditions, certain *JAZ* members are phosphorylated by the MAPK cascade, leading to enhanced stability and the shutdown of JA signaling, thereby switching the plant toward defense-prioritized growth. This allows distinct members to respond to multiple independent input signals such as drought, herbivory, and mechanical wounding (Wang et al., 2024; Hann et al., 2024; Li et al., 2024b; Zhao and Liu, 2023).

In summary, through molecular mechanisms such as gene duplication, alternative splicing, and domain shuffling, the *TIFY* gene family has greatly enriched the physicochemical properties and domain architectures of its proteins. This diversity is directly translated into functional differences in protein stability, interaction specificity, subcellular localization, and regulatory modes, thereby enabling the *TIFY* family to serve as a highly plastic regulatory platform that precisely coordinates multiple signaling networks ranging from plant development to stress responses.

### 4.5 Distribution characteristics of the *cis*-acting elements in the promoters of the *TIFY* gene family in *Malus sieversii* and their potential association with stress responses

The results of the promoter analysis of *MsTIFY* genes indicated that light-responsive elements (e.g., G-box and GT1 motif), hormone-responsive elements (e.g., the methyl jasmonate-responsive CGTCA motif and the salicylic acid-responsive TCA element), abiotic stress-responsive elements (e.g., the drought-inducible MBS and the low-temperature-responsive LTR), and dehydration-responsive elements (DRE) are widely distributed in the promoters of *TIFY* genes (Chattopadhyay et al., 1998; Toledo-Ortiz et al., 2014; Narusaka et al., 2003; Lescot et al., 2002; Kodackattumannil et al., 2023). The enrichment of these elements suggests that *TIFY* gene expression is likely under the coordinated regulation of light signals, phytohormone signals, and multiple environmental stress cues (Qing et al., 2025). Notably, the types and numbers of *cis*-acting elements differ among subfamily members (Singh and Mukhopadhyay, 2021; Zhang et al., 2015). Taking low-temperature stress as an example, significantly upregulated genes such as *MsJAZ11*, *MsJAZ12*, and *MsJAZ14* are enriched in LTR, DRE core, and STRE elements, conforming to the classical cold-acclimation CBF/DREB regulatory cascade (Liu et al., 1998). In contrast, the significantly downregulated *MsZML11*, *MsZML12*, and *MsZML13* contain a few ABRE motifs but completely lack LTR and DRE elements, which likely explains their inability to effectively mount a cold response. Under salt stress, the promoters of the leaf-upregulated genes, including *MsJAZ33*, *MsJAZ34*, *MsJAZ26*, *MsJAZ2*, and *MsJAZ6*, commonly harbor salt/drought-related elements such as ABRE, MYB, and Myc (Rubio and Pérez, 2025; Abe et al., 2003). On the other hand, *MsJAZ33* is downregulated in the roots: although its promoter contains ABRE motifs, it lacks root-specific activating elements, and we speculate that unknown repressive motifs (e.g., Unnamed motifs) may confer tissue-specific negative regulation. Under drought stress, ABA signaling occupies a central position: the leaf-upregulated genes (e.g., *MsJAZ33*, *MsJAZ26*, and *MsJAZ2*) carry multiple ABRE and G-box elements, supporting ABA-dependent drought responses (Fujita et al., 2011; Li et al., 2022). Conversely, the leaf-downregulated *MsTIFY1*, *MsJAZ11*, and *MsJAZ12* possess only a few ABRE motifs at markedly lower abundance and lack MYB binding sites and STRE elements, which may prevent the formation of effective transcriptional activation complexes. The composition of the *cis*-acting elements in the promoters of *M. sieversii TIFY* genes and their response characteristics to different abiotic stresses support a correlation between promoter architecture and gene function. It should be emphasized that bioinformatics-based *cis*-element prediction can only provide clues to potential binding sites: confirmation of their transcriptional regulatory functions still relies on molecular approaches such as yeast one-hybrid assays, electrophoretic mobility shift assays (EMSA), and promoter serial deletion experiments.

## 5 Conclusion

In *M. sieversii*, a total of 57 *MsTIFY* genes were identified, distributed across 21 chromosomes and classified into four distinct subgroups. This study systematically analyzed the physicochemical properties, phylogenetic relationships, conserved motifs, *cis*-acting regulatory elements, and synteny of the MsTIFY proteins. Subcellular localization predictions indicated that MsTIFY proteins are expressed in the nucleus. Expression profiling, based on transcriptomic data and validated by qRT-PCR, revealed specific response patterns of *MsTIFY* genes to abiotic stresses. Notably, *MsJAZ2* and *MsJAZ26* exhibited significant responsiveness to cold, salt, and drought stresses. These findings provide insight into the fundamental characteristics of the *MsTIFY* genes, thereby contributing to the improvement of stress tolerance in apple cultivars.

## Data Availability

The datasets presented in this study can be found in online repositories. The names of the repository/repositories and accession number(s) can be found in the article/**Supplementary Material**.
